# A learning based impedance control strategy implemented on a soft prosthetic wrist in joint-space

**DOI:** 10.3389/frobt.2025.1665267

**Published:** 2025-09-22

**Authors:** Shifa Sulaiman, Francesco Schetter, Ebrahim Shahabi, Fanny Ficuciello

**Affiliations:** 1 Department of Information Technology and Electrical Engineering, Università degli Studi di Napoli Federico II, Naples, Italy; 2 Cognitive Robotics Department of Engineering, Delft University of Technology, Delft, Netherlands

**Keywords:** prosthetic hand, euler-Bernoulli beam, euler, Lagrange method, soft robotics, impedance control

## Abstract

The development of advanced control strategies for prosthetic hands is essential for improving performance and user experience. Soft prosthetic wrists pose substantial control challenges due to their compliant structures and nonlinear dynamics. This work presents a learning-based impedance control strategy for a tendon-driven soft continuum wrist, integrated with the PRISMA HAND II prosthesis, aimed at achieving stable and adaptive joint-space control. The proposed method combines physics-based modeling using Euler-Bernoulli beam theory and the Euler-Lagrange approach with a neural network trained to estimate unmodeled nonlinearities. Simulations achieved a Root Mean Square Error (RMSE) of $3.04\times 10^{-4}$ rad and a settling time of 3.1 s under nominal conditions. Experimental trials recorded an average RMSE of $2.7\times 10^{-2}$ rad and confirmed the controller’s ability to recover target trajectories under unknown external forces. The method supports compliant interaction, robust motion tracking, and trajectory recovery, positioning it as a viable solution for personalized prosthetic rehabilitation. Compared to traditional controllers like Sliding Mode Controller (SMC), Model Reference Adaptive Controller (MRAC), and Model Predictive Controller (MPC), the proposed method achieved superior accuracy and stability. This hybrid approach successfully balances analytical precision with data-driven adaptability, offering a promising pathway towards intelligent control in next-generation soft prosthetic systems.

## 1 Introduction

The development of prosthetic hands has significantly advanced over the years, yet achieving natural and precise control remains a challenge. The main challenge in controlling soft continuum prosthetic hands stems from their inherent flexibility, which requires highly precise and adaptive control to execute a wide range of tasks effectively. Traditional control methods struggle to manage the non-linear dynamics and varying stiffness of these prosthetics. By integrating Neural Network (NN) into a control framework, it is possible to achieve a more responsive and intelligent system that can learn from interactions and adjust its behavior accordingly, thereby improving the overall functionality of the prosthetic hand (Gohari et al., 2025). The challenges associated with prosthetic hand motions include issues related to compliance, stability, and the ability to perform a wide range of tasks with varying degrees of precision. The proposed NN-based impedance controller aims to address these shortcomings by leveraging machine learning techniques to optimize the control parameters in real-time, allowing for a more fluid and natural user experience. In this work, we employ an NN-based impedance controller that aims to bridge this gap by leveraging the adaptability and learning capabilities of NNs to enhance the control of soft continuum prosthetic hands.

The evolution of prosthetic hand technologies has witnessed a growing shift towards anthropomorphic design principles that emphasize dexterity, adaptability, and user-centered control Sulaiman et al. (2024). Among these developments, tendon-driven soft continuum wrists have emerged as a promising solution due to their inherent compliance, lightweight structure, and ability to mimic the nuanced mobility of a human wrist. Such continuum designs can enhance the functional range of prosthetic hands and enable smoother, more intuitive manipulation of objects. However, exploiting their full potential requires advanced control strategies that account for the nonlinearities introduced by tendon elasticity, joint flexibility, and external disturbances.

Traditional position-based controllers often fall short in regulating movements within continuum mechanisms, as they assume rigid-link dynamics and fail to accommodate the variable mechanical impedance of soft structures. Impedance control, which modulates the dynamic relationship between force and motion, offers a compelling alternative by introducing compliant behavior that is crucial for safe and adaptable interaction with uncertain environments. Nonetheless, defining precise impedance parameters in systems characterized by nonlinear dynamics remains a significant challenge, particularly when implemented in real-time and under unpredictable loading conditions.

To address this gap, the current research proposes a learning-based impedance controller for a soft continuum tendon driven wrist attached to a PRISMA HAND II prosthesis. The kinematic model of the wrist is developed using the Euler–Bernoulli beam theory, capturing the bending behavior of the compliant structure, while the dynamic model is formulated via the Euler–Lagrange approach to account for system inertia and actuator influence. An NN is integrated within the control loop to estimate the nonlinear components of the impedance model, thereby enhancing the controller’s ability to compensate for unmodeled disturbances and time-varying system dynamics. This study further substantiates the controller’s effectiveness through detailed simulation studies and hardware testing. Evaluations focus on key performance metrics such as Root Mean Square Error (RMSE), steady-state error, and settling time, offering a comprehensive view of the controller’s ability to ensure accurate and responsive motion regulation. A comparison study of the proposed controller with similar controllers are carried out to showcase the advantages of the proposed controller. By combining physics-based modeling with data-driven learning, this work contributes to the advancement of hybrid control strategies that bridge analytical rigor with adaptability, paving the way toward more intelligent and intuitive prosthetic systems.

In this manuscript, the term soft robotics refers specifically to the tendon-driven prosthetic wrist system characterized by its compliant materials and continuum-like structure. Unlike rigid robotic mechanisms, this system integrates flexible tendons, elastic springs, and segmented discs that enable smooth, adaptive motion, and elastic deformation. The soft nature of the wrist is captured through its nonlinear dynamic behavior and modeled using Euler–Bernoulli beam theory, reflecting the challenges of controlling a compliant, continuum-based actuator. The organization of this paper is as follows: Section 2 reviews the current state of control strategies in prosthetic systems and highlights the limitations motivating this work. Section 3 introduces the proposed impedance control framework, including the mechanical design, mathematical modeling, and neural network integration. Section 4 presents the results of simulation studies conducted under varied mechanical and force conditions. Section 5 reports on experimental validation, showcasing real-world robustness of the controller. Section 6 compares the proposed approach with established control strategies such as Sliding Mode Controller (SMC), Model Reference Adaptive Controller (MRAC), and Model Predictive Controller (MPC). Finally, Section 7 summarizes the findings and outlines future directions for enhancing system performance and user personalization.

## 2 State of art

Advancements in robotics and human-machine interfaces have paved the way for the development of prosthetic systems that are not only functional but also adaptive and responsive to dynamic environments. In particular, soft prosthetic devices have gained attention for their ability to interact safely and comfortably with biological tissues, offering enhanced compliance and reduced mechanical impedance. However, controlling these devices in a way that mirrors natural joint behavior remains a significant challenge. This paper explores a novel approach that integrates a learning-based impedance control strategy within a soft prosthetic wrist, focusing on joint-space coordination to emulate human-like movements. The proposed method leverages machine learning algorithms to fine-tune impedance parameters in real-time, adapting to varying conditions and user intentions. By embedding intelligence directly into the control architecture, the system achieves a more nuanced and personalized response to external forces and user input. This innovation not only enhances motion fidelity and responsiveness but also holds promise for broad applications in wearable robotics and rehabilitation technologies. The implementation highlights a shift toward smarter, more intuitive prosthetic solutions that bridge the gap between mechanical performance and human adaptability.

Esquivel-Ortiz (2021) focused on enhancing grasp stability in upper-limb prosthetics. The work introduced an impedance control algorithm that dynamically adjusted to uncertainties such as object friction and contact points. Using the SynGrasp simulation environment, the study modeled various grasping configurations and evaluated the stability of grasps before and after perturbations. The results demonstrated improved grasp quality and adaptability under external disturbances. However, the research highlighted a gap in real-time implementation and the need for hardware validation to confirm simulation outcomes. Ferrante (2023) proposed a novel framework called AIC-UP that decodes human motor intent, including joint position, stiffness, and damping from surface Electromyography (EMG) signals. The system incorporated muscle-tendon unit models to estimate joint impedance and implemented these estimates on a simulated 1-DoF prosthetic wrist. Simulation results showed better control over both kinematics and impedance, but the study acknowledged limitations in decoding accuracy due to the noisy nature of EMG signals. The authors pointed out the need for improved signal processing and real-world testing to bridge the gap between simulation and practical deployment.

Wang et al. (2022) developed a prosthetic bionic hand system that combined myoelectric pattern recognition with adaptive control strategies. Their system used linear discriminant analysis (LDA) to classify sEMG signals and control a five-fingered prosthetic hand with linear servo motors. While not a pure impedance controller, the system incorporated compliance through mechanical design and feedback loops. The prosthetic hand achieved an average classification accuracy of 96.59% and performed well in grasping tests involving 15 objects of varying shapes and sizes. The study emphasized the need for integrating impedance modulation to further enhance adaptability and reduce cognitive load on users during dynamic tasks. In the domain of soft robotics, Mazare et al. (2022) proposed an Adaptive Variable Impedance Control (AVIC) strategy for a modular soft robot manipulator. The controller was designed using an adaptive back-stepping sliding mode approach and implemented in configuration space to handle model uncertainties and external forces. The system was benchmarked against sliding mode and inverse dynamics PD controllers, showing superior performance in stabilizing position errors and mitigating external disturbances. Despite its effectiveness, the study noted the complexity of tuning multiple control parameters and the lack of experimental validation on physical soft robotic platforms.

A hybrid impedance-admittance control strategy was explored by Rhee et al. (2023) to improve manipulator performance in environments with varying stiffness. Although the study focused on rigid manipulators, its findings are relevant to soft robotics due to the shared need for compliance. The controller dynamically switched between impedance and admittance modes based on environmental feedback, achieving better stability and accuracy in both simulation and physical experiments. The authors suggested that future work should explore how such hybrid strategies could be adapted for soft-bodied systems, where contact dynamics are more complex and less predictable. A study by Ferrante et al. (2024) introduced the AIC-UP framework, which estimates joint stiffness and damping from surface EMG signals using muscle-tendon models. Implemented on a simulated prosthetic wrist, the controller demonstrated superior robustness to muscle coactivation compared to NN-based kinematic decoders. However, the study acknowledged limitations in decoding accuracy due to EMG signal variability and emphasized the need for real-time hardware validation.

Shi et al. (2025) proposed a bio-signals-free control system for prosthetic hands using imitation learning, bypassing traditional EMG-based methods. Their system used a wrist-mounted camera and tactile sensors to autonomously grasp and release objects. The model, trained on a small dataset of human demonstrations, achieved over 95% success in real-world grasping tasks. However, the study noted the need for broader generalization across users and object types. Mora et al. (2024) presented a low-cost, real-time system for recognizing nine common grasping postures using sEMG signals and a machine learning approach. By extracting just two features from Myo armband data and applying a GPU-optimized multi-layer perceptron, the model achieved a 73% recognition accuracy across subjects. The method offered a robust, efficient solution for prosthetic hand control and human–robot interaction. García-Ortíz et al. (2024) introduced a model-based predictive control (MBPC) strategy to improve dexterity and energy efficiency of prosthetic hands. The work applied linear identification techniques to model the dynamic behavior of prosthetic fingers, which is then used to implement a generalized predictive control (GPC) algorithm. Experimental validation on a test bench showed that the proposed control system can accurately manage finger positions, anticipate future movements, and minimize power consumption.

Lai et al. (2025) introduced a 3D-printed hydrogel-based sEMG electrode array for prosthetic control. The soft, stretchable electrodes improved skin conformity and signal fidelity, enabling more accurate decoding of hand gestures. Integrated with an AI-based classifier, the system achieved real-time control of a prosthetic hand. Despite its success, the authors highlighted challenges in long-term durability and signal drift under motion artifacts. Wu et al. (2023) developed an adaptive impedance control algorithm for dexterous hand-object interaction. Their admittance-based controller adjusted parameters based on object dynamics and was deployed on a multi-fingered robotic hand. Experimental results showed effective force regulation across objects with varying stiffness. However, the study lacked real-world prosthetic integration and called for further testing in unstructured environments. Khan and Li (2024) proposed a discrete-time sliding mode impedance controller for pneumatic soft robots. Their controller regulated overshoot and vibration during deactuation, a common issue in soft actuators. Tested on a 6-chambered parallel soft robot, the system outperformed traditional SMCs in damping and settling time. The authors noted the need for real-time embedded implementation and broader task generalization.

Stölzle et al. (2024) combined Electroencephalogram (EEG) based motor imagery with impedance control to guide soft robots. Their system used a Cartesian impedance controller to translate brain signals into end-effector motion. Despite using only three EEG channels, users achieved 66% task success in setpoint regulation. The study demonstrated the feasibility of brain-controlled soft robots but acknowledged the low signal-to-noise ratio of EEG and the need for improved classification accuracy. Mountain et al. (2024) introduced a grasping force adaptation algorithm for a cable-driven prosthetic hand using Youla-parameterization and iterative learning control. The impedance controller adjusted grasp stiffness based on tactile feedback, improving performance across object weights. While effective, the method required extensive training data and computational resources, limiting its real-time applicability.

A study by Gao et al. (2025) reviewed human-machine interfaces for soft robotic systems, emphasizing the role of impedance control in enhancing interaction safety and adaptability. The paper surveyed recent advances in sensor integration, algorithmic control, and wearable interfaces. It identified a research gap in multi-modal sensor fusion and the need for standardized benchmarking in soft prosthetic applications. Jadav and Palanthandalam-Madapusi (Jadav and Palanthandalam-Madapusi, 2024) proposed a variable impedance control algorithm that adapts to divergent force fields without relying on Jacobian inversion. Tested on a 7-DOF KUKA arm and a simulated human arm, the controller demonstrated faster relearning and improved stability. While promising, the method’s application to soft or prosthetic systems remains unexplored, presenting a clear direction for future work. A review by Rajashekhar and Prabhakar (2025) explored human-robot interaction in soft robotics, highlighting impedance control as a key enabler of safe collaboration. The paper discussed input/output modalities, actuator design, and ethical considerations. It emphasized the lack of standardized HRI protocols for soft prosthetics and called for interdisciplinary research bridging materials science, control theory, and user-centered design.

Several studies have explored the use of NN and impedance control in prosthetics. For instance, a chronological overview of control strategies for prosthetic hands highlights the application of NN in estimating muscular contraction levels and controlling impedance parameters was demonstrated in (Naidu et al., 2008). Additionally, research on soft-synergy prosthetic hands Basumatary and Hazarika (2020) has demonstrated the potential of NN-based controllers in improving force modulation and grasp performance. Another study (Portnova-Fahreeva et al., 2023) introduced an autoencoder-based myoelectric controller, showcasing the effectiveness of NN in managing high-dimensional prosthetic hand systems. These studies collectively underscore the potential of NN-based impedance controllers in enhancing the functionality and user experience of soft continuum prosthetic hands.

The exploration of utilizing an NN-based impedance controller for the regulation of movements in a soft continuum prosthetic hand is driven by the need for enhanced dexterity and adaptability in prosthetic devices. Traditional control methods often fail to provide the nuanced control required for complex tasks, particularly in dynamic environments. By integrating NNs into the control framework, it is possible to achieve a more responsive and intelligent system that can learn from interactions and adjust its behavior accordingly, thus improving the overall functionality of the prosthetic hand. The problem statement focuses on the limitations of existing control strategies for soft continuum prosthetic hands, which often struggle to replicate the intricate movements of a natural hand. These challenges include issues related to compliance, stability, and the ability to perform a wide range of tasks with varying degrees of precision. The proposed NN-based impedance controller aims to address these shortcomings by leveraging machine learning techniques to optimize the control parameters in real-time, allowing for a more fluid and natural user experience.

## 3 Methodology

**FIGURE 1:**
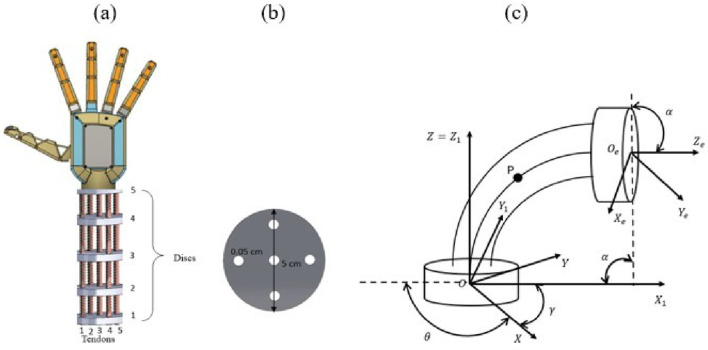
Soft wrist section **(a)** Conceptual design of wrist section attached to hand **(b)** Dimension of disc **(c)** Bending structure of wrist section.

The design of the proposed soft wrist segment, as detailed in Sulaiman et al. (2024a) is connected to a prosthetic hand named ’PRISMA HAND II’ (Liu et al., 2019), comprises five rigid discs, five springs, and five flexible tendons, as depicted in Figure 1a along with rigid disc dimensions in Figure 1b. Figure 1c illustrates the bending configuration of the soft wrist segment with length l , radius r , a bending angle of α , division angle with respect to horizontal axes θ , and the rotation angle of the bending plane, γ .

**FIGURE 2:**
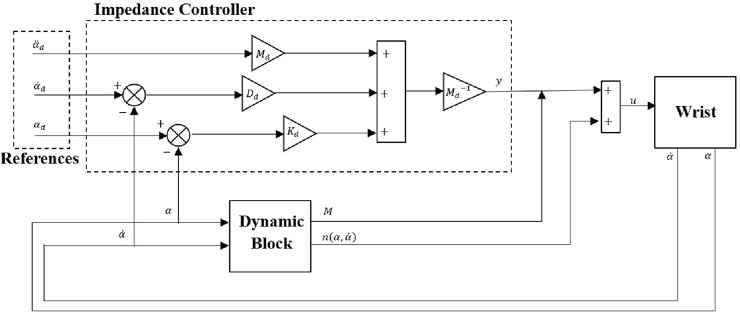
Control scheme.

The springs and tendons are integrated into the rigid discs and secured to a solid platform. The positioning of the end effector in relation to the curvature of the wrist is determined through the principles of Euler-Bernoulli beam theory, as referenced in (He et al., 2013). Dynamic model of the wrist section was determined using Lagrange equation as cited in (Liu et al., 2019) and used in Impedance control strategy. Desired bending angles (α) , rate of bending angles $\left(\dot{\alpha }\right)$ , and derivative of rate of bending angles $\left(\ddot{\alpha }\right)$ were fed to the impedance controller as shown in Figure 2. The impedance controller was designed assuming a predominantly capacitive environment, where stiffness plays a central role in interaction dynamics.

The transformation matrix (T) , which defines the pose of disc five relative to the base disc one, is given in Equation 1

$$T=\left[\begin{array}{cc} R & P\\ 0 & 1 \end{array}\right]$$ (1)

where rotation matrix, R is obtained as given in Equation 2

$$R=Rot\left(z,\gamma \right)Rot\left(y,\alpha \right)Rot\left(z,-\gamma \right)=\left[\begin{array}{ccc} c^{2}\gamma c\alpha +s^{2}\gamma & c\gamma s\gamma c\alpha -c\gamma s\gamma & c\gamma s\alpha \\ c\gamma s\gamma c\alpha -c\gamma s\gamma & s^{2}\gamma c\alpha +c^{2}\gamma & s\gamma s\alpha \\ -c\gamma s\alpha & -s\gamma c\alpha & c\alpha \end{array}\right]$$ (2)

where s represents the arc-length parameter of the segments ( s=0 corresponds to the base disk and s=l denotes the end disk). Translational matrix P is given in Equation 3.

$$P={\left[\begin{array}{ccc} x & y & z \end{array}\right]}^{T}=\left[\begin{array}{ccc} \frac{l}{\alpha }\left(1-\cos\frac{s\alpha }{l}\right)\cos\gamma & \frac{l}{\alpha }\left(1-\cos\frac{s\alpha }{l}\right)\sin\gamma & \frac{l}{\alpha }\sin\frac{s\alpha }{l} \end{array}\right]$$ (3)

The kinetic energy of the wrist section’s motion is determined by computing the time derivatives of the position vectors provided in Equation 3. The corresponding velocity expressions are given as follows:

$$\left\{\begin{array}{c} \begin{array}{cc} \frac{dx}{dt} & =\frac{1}{\alpha }\left[s\sin\frac{s\alpha }{l}\cos\gamma -\frac{l}{\alpha }\left(1-\cos\frac{s\alpha }{l}\right)\cos\gamma \right]\frac{d\alpha }{dt}\\ & \;-\frac{1}{\alpha }\left(1-\cos\frac{s\alpha }{l}\right)\sin\gamma \frac{d\gamma }{dt} \end{array}\;\\ \begin{array}{cc} \frac{dy}{dt} & =\frac{1}{\alpha }\left[s\sin\frac{s\alpha }{l}\sin\gamma -\frac{l}{\alpha }\left(1-\cos\frac{s\alpha }{l}\right)\sin\gamma \right]\frac{d\alpha }{dt}\\ & \;+\frac{1}{\alpha }\left(1-\cos\frac{s\alpha }{l}\right)\cos\gamma \frac{d\gamma }{dt} \end{array}\;\\ \begin{array}{cc} \frac{dz}{dt} & =\frac{1}{\alpha }\left(s\cos\frac{s\alpha }{l}-\frac{l}{\alpha }\sin\frac{s\alpha }{l}\right)\frac{d\alpha }{dt} \end{array}\; \end{array}\right.$$ (4)

The kinetic energy of the primary backbone (central tendon) of the soft wrist, denoted as $E_{k1}$ , can be expressed as follows:

$$E_{k1}=\frac{1}{2}\int_{0}^{l}\left[\begin{array}{c} {\left(\frac{dx}{dt}\right)}^{2}+{\left(\frac{dy}{dt}\right)}^{2}+{\left(\frac{dz}{dt}\right)}^{2} \end{array}\right]\rho Ads$$ (5)

Here, ρ and A denote the density and cross-sectional area of the wrist section, respectively. By substituting Equation 4 into Equation 5, the kinetic energy is obtained as shown in Equation 6.

$$E_{k1}=\frac{1}{6}m_{1}l^{2}{\left(\frac{d\alpha }{dt}\right)}^{2}K_{1}+\frac{1}{8}m_{1}l^{2}{\left(\frac{d\gamma }{dt}\right)}^{2}K_{2}$$ (6)

Here, $m_{1}$ represents the mass of the primary backbone, while $K_{1}$ and $K_{2}$ are the kinetic energy equivalent factors. The kinetic energy coefficients $K_{1}$ and $K_{2}$ are determined as shown in Equations 7, 8.

$$K_{1}=\left(\alpha^{3}+6\alpha -12\sin\alpha +6\alpha \cos\alpha \right)/\alpha^{5}$$ (7)

$$K_{2}=\left(6\alpha 8\sin\alpha +\sin2\alpha \right)/\alpha^{3}$$ (8)

From Equations 7, 8, the coefficients $K_{1}$ and $K_{2}$ can be expressed as functions of the bending angle α . These equations can be further simplified using a least squares fit, as shown in Equations 9, 10.

$$K_{1}=-0.00426\alpha^{2}-0.00277\alpha +0.15085$$ (9)

$$K_{2}=-0.05567\alpha^{3}+0.2328\alpha^{2}+0.006216\alpha -0.00406$$ (10)

The transformation between Cartesian space and joint space can be expressed as given in Equation 11:

$$\left\{\begin{array}{c} q_{1}=r\alpha \cos\left(\gamma \right)\;\\ q_{2}=r\alpha \cos\left(-\gamma +\theta \right)\;\\ q_{3}=r\alpha \cos\left(\gamma +\theta \right)\; \end{array}\right.$$ (11)

Here, $q_{i}\;\left(i=1,2,3\right)$ denotes the length of each driving wire, and r represents the distance from each secondary backbone to the primary backbone, assuming the secondary backbone tendons are equidistant from the primary backbone. The angular separation is given by $\theta =\frac{2\pi }{3}$ . The driving velocities are obtained by differentiating Equation 11, and are expressed as given in Equation 12:

$$\left\{\begin{array}{c} \frac{dq_{1}}{dt}=r\cos\left(\gamma \right)\frac{d\alpha }{dt}-r\alpha \sin\left(\gamma \right)\frac{d\gamma }{dt}\\ \frac{dq_{2}}{dt}=r\cos\left(-\gamma +\theta \right)\frac{d\alpha }{dt}+r\alpha \sin\left(-\gamma +\theta \right)\frac{d\gamma }{dt}\\ \frac{dq_{3}}{dt}=r\cos\left(\gamma +\theta \right)\frac{d\alpha }{dt}-r\alpha \sin\left(\gamma +\theta \right)\frac{d\gamma }{dt} \end{array}\right.$$ (12)

The secondary backbone consisted of four tendons. However, for analytical purposes, the tendons located on each side during motion are treated as a single tendon. For instance, when the tendons rotate in the direction of ulnar deviation, tendons four and five are considered as one, while tendons one and two are treated as two distinct tendons. The total kinetic energy $E_{k2}$ of the secondary backbone, composed of four tendons, is given in Equation 13.

$$E_{k2}=E_{\mathrm{k11}}+E_{\mathrm{k22}}$$ (13)

where $E_{\mathrm{k11}}$ = $E_{k1}$ and the second component, $E_{\mathrm{k22}}$ arises from the driven kinetic energy as given in following Equation 14:

$$E_{\mathrm{k22}}=\frac{1}{2}m_{1}\left[\begin{array}{c} {\left(\frac{dq_{1}}{dt}\right)}^{2}+{\left(\frac{dq_{2}}{dt}\right)}^{2}+{\left(\frac{dq_{3}}{dt}\right)}^{2} \end{array}\right]$$ (14)

Substituting Equation 12 in Equation 14, we obtain the following Equations 15–18

$$E_{k2}=\frac{1}{2}m_{2}\left[{\left(\frac{d\alpha }{dt}\right)}^{2}K_{3}+\frac{d\alpha }{dt}\frac{d\gamma }{dt}K_{4}+{\left(\frac{d\gamma }{dt}\right)}^{2}K_{5}\right]$$ (15)

$$K_{3}=r^{2}\left[\begin{array}{c} \cos^{2}\left(\gamma \right)+\cos^{2}\left(-\gamma +\theta \right)+\cos^{2}\left(\gamma +\theta \right) \end{array}\right]$$ (16)

$$K_{4}=r^{2}\alpha \left[\begin{array}{c} -\sin\left(2\gamma \right)+\sin\left(2\left(-\gamma +\theta \right)\right)-\sin\left(2\left(\gamma +\theta \right)\right) \end{array}\right]$$ (17)

$$K_{5}=r^{2}\alpha^{2}\left[\begin{array}{c} \sin^{2}\left(\gamma \right)+\sin^{2}\left(-\gamma +\theta \right)+\sin^{2}\left(\gamma +\theta \right) \end{array}\right]$$ (18)

where $m_{2}$ is the mass of the secondary backbone and $K_{3}$ , $K_{4}$ and $K_{5}$ are kinetic energy equivalent factors. The kinetic energy of the discs can be obtained as given in Equation 19:

$$E_{k3}=\frac{1}{2}m_{3}{\left(\frac{d\alpha }{dt}\right)}^{2}K_{6}+\frac{1}{2}m_{3}{\left(\frac{d\gamma }{dt}\right)}^{2}K_{7}$$ (19)

Here, $m_{3}$ denotes the mass of a disk, and $K_{6}$ and $K_{7}$ are the kinetic energy equivalent factors. If the parameters n and h are known, the coefficients $K_{6}$ and $K_{7}$ can be expressed as functions of the bending angle α . Assuming n=5 and h=15 mm , the expressions for $K_{6}$ and $K_{7}$ can be simplified using a least squares fit, as shown in Equations 20, 21:

$$K_{6}=\left(-0.00043\alpha^{2}-0.00031\alpha +0.01435\right)/2$$ (20)

$$K_{7}=\left(-0.00394\alpha^{3}+0.01575\alpha^{2}+0.00131\alpha -0.00047\right)/2$$ (21)

For a continuum robot, the total potential energy consists of two components: elastic potential energy and gravitational potential energy. In this context, the gravitational component is considered negligible in comparison to the elastic potential energy. The elastic energy $E_{p}$ , associated with the wrist section and characterized by Young’s modulus E and area moment of inertia I , is given in Equation 22:

$$E_{p}=\frac{2EI}{l}\alpha^{2}$$ (22)

The Lagrange equation governing the dynamics of the wrist section is expressed as in Equation 23:

$$\frac{d}{dt}\frac{\partial E_{k}}{\partial \dot{p_{j}}}-\frac{\partial E_{k}}{\partial p_{j}}+\frac{\partial E_{p}}{\partial p_{j}}=Q_{j},\left(j=1,2\right)$$ (23)

where $Q_{j}$ represents the generalized force of system, $E_{k}=E_{k1}+E_{k2}+E_{k3}$ , $p_{1}=\alpha$ and $p_{2}=\gamma$ . The dynamical Eq. of the wrist is obtained as given in Equation 24:

$$\begin{array}{ll} \left[\begin{array}{cc} M_{11} & M_{12}\\ M_{21} & M_{22} \end{array}\right]\left[\begin{array}{c} \ddot{\alpha }\\ \ddot{\gamma } \end{array}\right] & +\left[\begin{array}{ccc} C_{11} & C_{12} & C_{13}\\ C_{21} & C_{22} & C_{23} \end{array}\right]\left[\begin{array}{c} {\dot{\alpha }}^{2}\\ \dot{\alpha }\dot{\gamma }\\ {\dot{\gamma }}^{2} \end{array}\right]+\left[\begin{array}{cc} K_{11} & K_{12}\\ K_{21} & K_{22} \end{array}\right]\\ & \;\left[\begin{array}{c} \alpha \\ \gamma \end{array}\right]=\left[\begin{array}{cc} D_{11} & D_{12}\\ D_{21} & D_{22} \end{array}\right]\left[\begin{array}{c} F_{1}\\ F_{2} \end{array}\right] \end{array}$$ (24)

where $M_{ij},C_{ij},K_{ij},D_{ij}$ are moment of inertia, Coriolis, stiffness, actuation matrix elements respect to each rotation angle. In the context of planar motion, where γ=0 , we determined the Equation of motion as given in Equation 25:

$$M\left(\alpha \right)\ddot{\alpha }+C\left(\alpha \right){\dot{\alpha }}^{2}+K\alpha =DF$$ (25)

where:

$$D=r\cos\left(\gamma \right)$$

$$K=\frac{4EI}{l}$$

$$C=-\frac{1}{6}\left(4m_{2}l^{2}\frac{\partial K_{1}}{\partial \alpha }\right)+3m_{2}\left(\frac{\partial K_{3}}{\partial \alpha }\right)+3m_{3}\left(\frac{\partial K_{6}}{\partial \alpha }\right)$$

$$M=\frac{1}{3}\left(4m_{2}l^{2}K_{1}+3m_{2}K_{3}+3m_{3}K_{6}\right)$$

Let us consider the following dynamic Equation 26 of a continuum wrist section with two sub sections with masses $m_{1}$ and $m_{2}$ respectively, Young’s modulus (E) , moment of inertia (I) , energy coefficients ( $K_{i}$ , i = 1,2,.6), and actuation force or torque applied to the soft prosthetic wrist system (u)

$$M\left(\alpha \right)\ddot{\alpha }+C\left(\alpha ,\dot{\alpha }\right)\dot{\alpha }+K\alpha =u-f_{\mathrm{ext}}$$ (26)

where $M\left(\alpha \right)=D_{11}^{-1}M_{11}\left(\alpha \right)$ , $C\left(\alpha ,\dot{\alpha }\right)=D_{11}^{-1}C_{11}\left(\alpha ,\dot{\alpha }\right)$ , $K=D_{11}^{-1}K_{11}$ . $M_{11}=\frac{1}{3}\left(4m_{1}l^{2}K_{1}+3m_{1}K_{3}+3m_{2}K_{6}\right)$ , $C_{11}=\frac{1}{6}\left(4m_{1}l^{2}\frac{\partial K_{1}}{\partial \alpha }+3m_{1}\frac{\partial K_{3}}{\partial \alpha }+3m_{2}\frac{\partial K_{6}}{\partial \alpha }\right)$ , $K_{11}=\frac{4EI}{l}$ , $D_{11}=R$ are the inertia matrix, Coriolis matrix, stiffness matrix, and actuation matrix of the system respectively. $f_{\mathrm{ext}}$ is the external force acting on system. A feedback linearization was achieved by choosing u as given in Equation 27:

$$u=M\left(\alpha \right)y+n\left(\alpha ,\dot{\alpha }\right)$$ (27)

where $n\left(\alpha ,\dot{\alpha }\right)=C\left(\alpha ,\dot{\alpha }\right)\dot{\alpha }+K\alpha$ .

$$M\left(\alpha \right)\ddot{\alpha }=M\left(\alpha \right)y-f_{\mathrm{ext}}$$ (28)

Equation 28 can be rewritten as given in Equation 29:

$$\ddot{\alpha }=y-M{\left(\alpha \right)}^{-1}f_{\mathrm{ext}}$$ (29)

**FIGURE 3:**
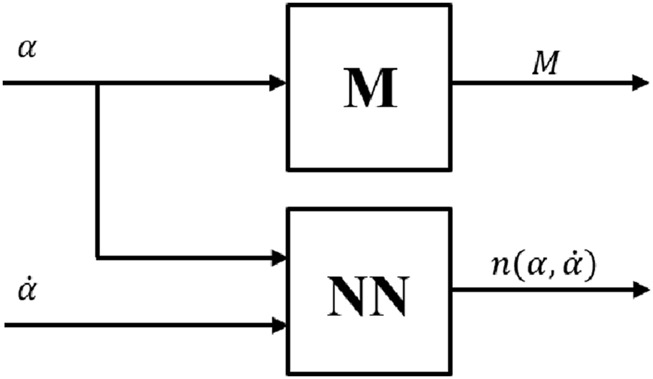
Dynamic block.

In order to achieve an impedance behavior, y was chosen as given in Equation 30:

$$y=M_{d}^{-1}\left(M_{d}{\ddot{\alpha }}_{d}+D_{d}\dot{\tilde{\alpha }}+K_{d}\tilde{\alpha }\right)$$ (30)

where $\tilde{\alpha }=\alpha_{d}-\alpha$ and $M_{d},D_{d},K_{d}$ are desired values of inertia, damping, and stiffness of the system at closed loop respectively. Equation 29 can be rewritten as given in Equations 31–33:

$$\ddot{\alpha }=M_{d}^{-1}\left(M_{d}{\ddot{\alpha }}_{d}+D_{d}\dot{\tilde{\alpha }}+K_{d}\tilde{\alpha }\right)-M{\left(\alpha \right)}^{-1}f_{\mathrm{ext}}$$ (31)

$$M_{d}\ddot{\alpha }=M_{d}{\ddot{\alpha }}_{d}+D_{d}\dot{\tilde{\alpha }}+K_{d}\tilde{\alpha }-M_{d}M{\left(\alpha \right)}^{-1}f_{\mathrm{ext}}$$ (32)

$$M_{d}\ddot{\tilde{\alpha }}+D_{d}\dot{\tilde{\alpha }}+K_{d}\tilde{\alpha }=F_{\mathrm{ext}}$$ (33)

where $F_{\mathrm{ext}}=M_{d}M{\left(\alpha \right)}^{-1}f_{\mathrm{ext}}$ . Equation 33 represents the error dynamics in a closed-loop system. When an external force is exerted on the system, it exhibits compliance. Once the external force is removed, the system returns to its original position, resulting in the error approaching zero. In our approach, we trained an NN to determine $n\left(\alpha ,\dot{\alpha }\right)$ to reduce the computational time during simulation and experimental studies. Figure 3 represents the detailed view of the dynamic block shown in Figure 2. Inertia matrix, M is dependent on α values. NN block receives α and $\dot{\alpha }$ as inputs and predicts $n\left(\alpha ,\dot{\alpha }\right)$ values.

## 4 Result and discussion

Simulation studies were conducted to determine the performance of the proposed controller in different scenarios. We have acquired the input-output dataset from a conventional impedance control scheme developed for the same wrist section. The data was obtained through simulation studies and experimental validations, and was subsequently used to train an NN for improved control performance. We utilised an NN with feed-forward back propagation configuration, trained using bending angles as the input and obtained $n\left(\alpha ,\dot{\alpha }\right)$ from the NN block.The NN architecture used in this study was selected based on empirical evaluation and prior experience with similar control tasks in soft robotics. A feedforward network with three hidden layers comprising 5, 7, and 10 neurons respectively was implemented, using the Levenberg-Marquardt backpropagation algorithm for training. This configuration was chosen to balance model complexity and training efficiency, ensuring sufficient capacity to capture the nonlinearities in the impedance model without overfitting. The choice of layer sizes reflected a balance between model expressiveness and computational efficiency, particularly given the real-time requirements of the prosthetic control system. While more complex architectures (e.g., deeper networks or convolutional layers) were considered, initial trials indicated diminishing returns in performance relative to increased training time and resource demands. Preliminary trials with shallower architectures (e.g., single or two-layer networks) resulted in higher RMSE and slower convergence, while deeper networks introduced unnecessary computational overhead without significant performance gains. The Levenberg-Marquardt backpropagation scheme was chosen for its rapid convergence properties in small-to medium-sized networks, making it well-suited for the regression tasks involved.

**FIGURE 4:**
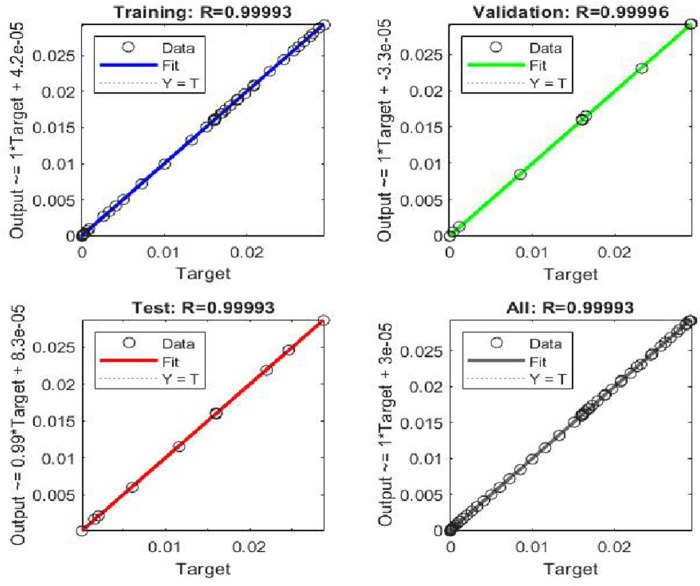
Regression graphs.

Tansig function and Levenberg Macquardt were used as the activation function and back propagation technique respectively. Regression scheme of the NN training is given in Figure 4. The regression scheme showcased an accuracy of 99.99% as evident from Figure 4.

**FIGURE 5:**
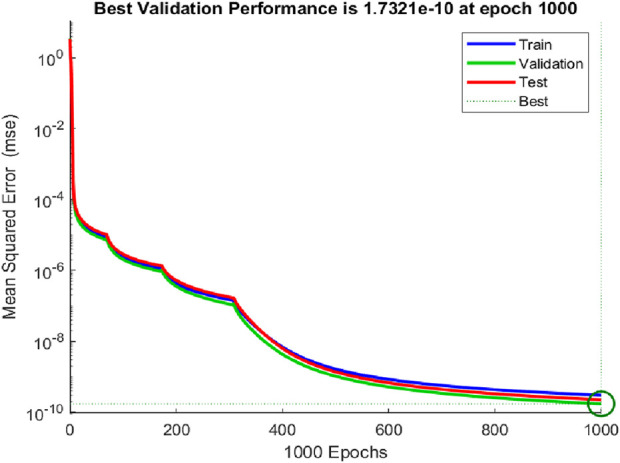
Performance graphs.

The performances of the NN with respect to training, validation, and test data are shown in Figure 5. Values of gradient and momentum (mu) were obtained as $1.08\times 10^{-14}$ and $1\times 10^{-12}$ respectively. The lower values of gradient and momentum showcased the successful convergence of the NN. Although a full hyperparameter search was not conducted due to computational constraints, the selected architecture consistently achieved high accuracy across training, validation, and test sets, with regression values exceeding 0.999 and minimal gradient and momentum values. These results indicated successful convergence and generalization.

**FIGURE 6:**
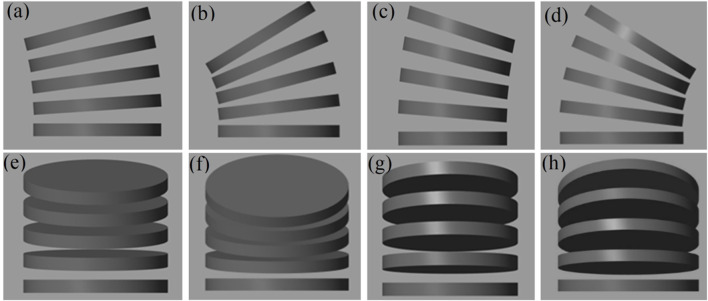
Motion of wrist **(a)** Radial-1 **(b)** Radial-2 **(c)** Ulnar-1 **(d)** Ulnar-2 **(e)** Flexion-1 **(f)** Flexion-2 **(g)** Extension-1 **(h)** Extension-2.

The wrist segment was considered to be flexing from its original position (without carrying prosthetic hand) to a final bending angle of 0.6 radians in all directions relative to the disc connected to the hand as shown in Figure 6.

**FIGURE 7:**
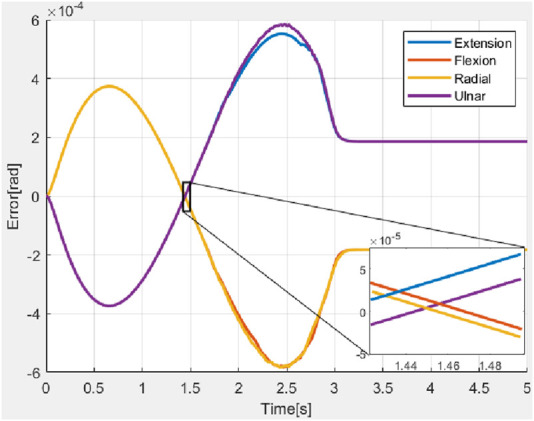
Error in bending angles during simulation.

The errors in deflections obtained during the simulations without the presence of external disturbances (nominal condition) are shown in Figure 7.

**FIGURE 8:**
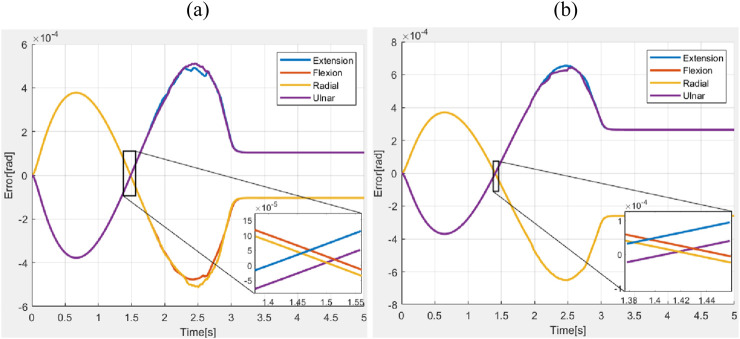
Wrist motions **(a)** Less stiffness **(b)** High stiffness.

Average values of RMSE, settling time, and steady state error were obtained as $3.04\times 10^{-4}\;rad$ , 3.1 s , and $1.25\times 10^{-4}\;rad$ respectively. To examine how changes in spring stiffness affect the system’s behavior, a simulation study was performed by varying the stiffness values within a ± 20% range of the nominal value as shown in Figures 8a,b. The average values of RMSE, settling time, and steady-state error for the reduced stiffness scenario were measured as $3.16\times 10^{-4}\text{ rad}$ , 3.2 s , and $1.33\times 10^{-4}\text{ rad}$ , respectively. Similarly, for the increased stiffness scenario, the average values of RMSE, settling time, and steady-state error were found to be $2.99\times 10^{-4}\text{ rad}$ , 3.1 s , and $1.10\times 10^{-4}\text{ rad}$ , respectively.

**FIGURE 9:**
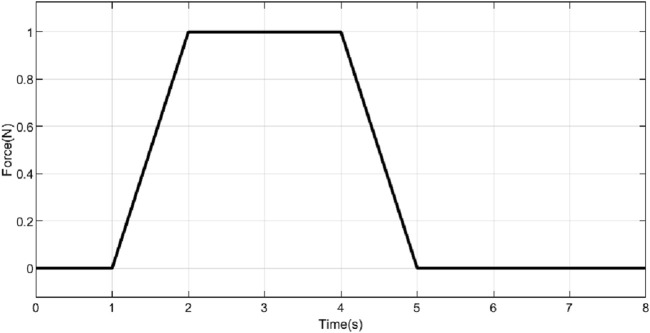
Constant force applied to prosthetic hand.

**FIGURE 10:**
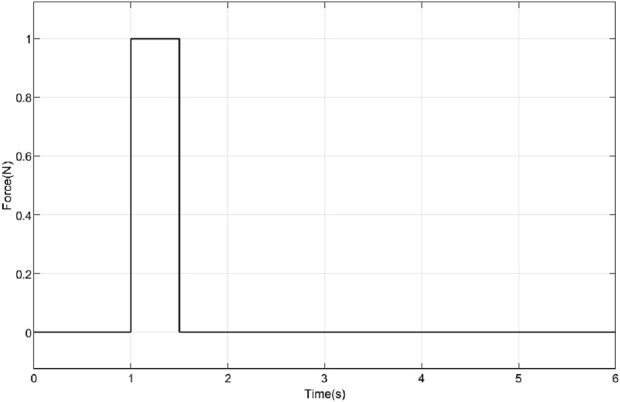
Impulse force applied to prosthetic hand.

**FIGURE 11:**
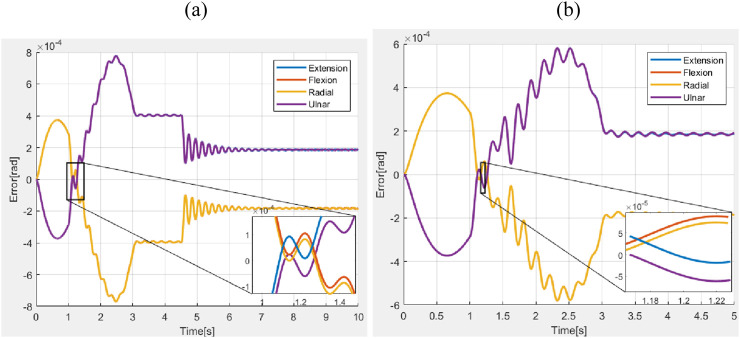
Error in wrist motions with hand **(a)** constant force **(b)** Impulse force.

Simulations were conducted to study the performance of the proposed controller in the presence of a constant force and a shock force as shown in Figures 9, 10 respectively. A constant force of 1 N is applied to the mid-section of the prosthetic hand, perpendicular to the direction of motion, for a duration of 2 s starting at t=2 s . Additionally, an impulsive force of 1 N is applied to the wrist section for a duration of 0.5 s starting at t=1 s . The errors obtained during the motions are shown in Figures 11a,b. The average values of RMSE, settling time, and steady-state error during the application of the constant force scenario were measured as $4.25\times 10^{-4}\text{ rad}$ , 8 s , and $2.04\times 10^{-4}\text{ rad}$ , respectively. Similarly, during the application of the impulsive force, the average values of RMSE, settling time, and steady-state error were found to be $3.87\times 10^{-4}\text{ rad}$ , 3.5 s , and $1.97\times 10^{-4}\text{ rad}$ , respectively.

**FIGURE 12:**
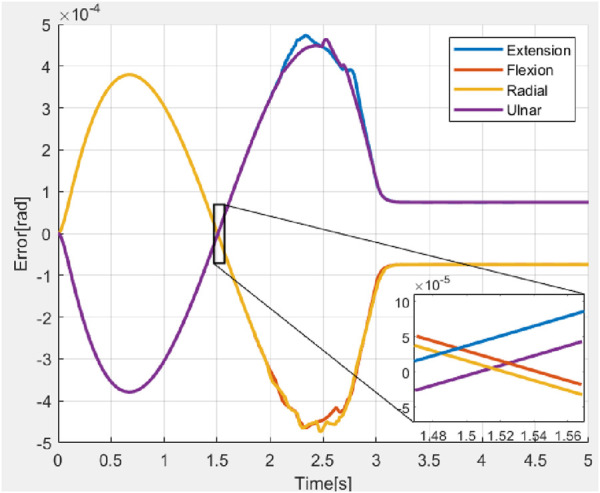
Wrist simulations with hand and an extra load.

A stability test was conducted by increasing the load on the hand by 50% to evaluate the controller’s robustness under higher external forces. The system’s behavior remained consistent with previous observations, maintaining stability and converging within approximately 3.7 s with RMSE and steady state errors of $3.45\times 10^{-4}\text{ rad}$ and $1.74\times 10^{-4}\text{ rad}$ respectively, as illustrated in Figure 12.

Across all the scenarios such as the nominal conditions, and stiffness variations, differences in performance measured in terms of RMSE, settling time, and steady-state error are minimal. This indicated that the chosen closed-loop parameters (as defined in Equation 33) were robust to variations in system parameters. Figure 11 explored the system’s response to external forces. In Figure 11a, a constant force of 1 N was applied (refer to Figure 9), while in Figure 11b, an impulsive force of 1 N was applied at 1 s (refer to Figure 10). In the first case, the ascending ramp influenced the transient phase of the error evolution (up to 3 s), while the constant force affected the steady-state behavior. Since the objective was to resist external disturbances, it is notable that during the constant phase, the error reached only $4\times 10^{-4}$ rad for an applied force of 1 N, a significant force for highly compliant systems making this an acceptable result. During the descending ramp, as the force returned to zero, the system entered an oscillatory state. This was expected, as the closed-loop system behaved like a mass-damper-spring system, and force variations naturally induced oscillations. In the second case, the oscillatory behavior persisted throughout the entire error evolution, due to the impulsive force applied during the transient phase at 1 s. In both cases, the steady state was not a fixed value but a permanent oscillatory condition caused by the selected closed-loop parameters. However, the oscillation was negligible. Although the control action reflected an oscillatory behavior, the amplitude was sufficiently low that it did not pose any risk to the actuators, especially considering that the control signal was filtered before being transmitted to the motors.

Table 1 presents a comparison of the RMSE, settling time, and steady-state error across different test scenarios. The normal scenario served as the baseline, with moderate values for all metrics. When the load on the hand is increased by 50% , the system exhibited a higher RMSE and steady-state error, along with a longer settling time, indicating reduced performance under heavier external forces. In contrast, increasing the stiffness slightly improved both RMSE and steady-state error, suggesting enhanced precision and control. Decreasing the stiffness leads to a marginal increase in RMSE and settling time, reflecting a slight degradation in performance. Under the constant force scenario, the system showed the highest RMSE and the longest settling time, highlighting the significant impact of sustained external disturbances on stability and accuracy. The impulsive force scenario also resulted in elevated RMSE and steady-state error, though the system recovered more quickly, as indicated by a shorter settling time compared to the constant force case. Overall, the controller demonstrated robustness across varying conditions, with performance variations aligning with the nature and intensity of the applied disturbances.

**TABLE 1:** Scenarios.

Scenario	RMSE	Settling time (s)	Steady-state error
Normal	$3.04\times 10^{-4}\;rad$	3.1	$1.25\times 10^{-4}\;rad$
Increased load	$3.45\times 10^{-4}\;rad$	3.7	$1.74\times 10^{-4}\;rad$
Increased stiffness	$2.99\times 10^{-4}\;rad$	3.1	$1.10\times 10^{-4}\;rad$
Decreased stiffness	$3.16\times 10^{-4}\;rad$	3.2	$1.33\times 10^{-4}\;rad$
Constant force	$4.25\times 10^{-4}\;rad$	8	$2.04\times 10^{-4}\;rad$
Impulsive force	$3.87\times 10^{-4}\;rad$	3.5	$1.97\times 10^{-4}\;rad$

### 4.1 Comparison with other controllers

The performance of the neuro-impedance controller was compared with other controllers developed for the wrist section. Results obtained using an SMC (Sulaiman et al., 2025b), an MRAC (Sulaiman et al., 2025a), and an MPC (Schetter et al., 2025) developed for the wrist section were compared with the results obtained using the proposed controller. RMSE, settling time and steady state error were compared to analyse the performances of the controllers as given in Table 2.

**TABLE 2:** Comparison study.

Parameters	SMC	MRAC	MPC	Impedance
RMSE	$6.0\times 10^{-3}\;rad$	$1.2\times 10^{-3}\;rad$	$2.1\times 10^{-3}\;rad$	$3.04\times 10^{-4}\;rad$
Settling time (s)	1.5	2.8	1.2	3.1
Steady state error	$1.3\times 10^{-3}\;rad$	$1.3\times 10^{-3}\;rad$	$0.4\times 10^{-5}\;rad$	$1.25\times 10^{-4}\;rad$

To ensure a meaningful comparison between control strategies, the cost function adopted in the MPC framework was carefully designed to balance tracking accuracy and control effort. The cost function is defined as:

$$J\left(z_{k}\right)=J_{x}\left(z_{k}\right)+J_{\Delta u}\left(z_{k}\right)+J_{\varepsilon }\left(z_{k}\right)$$ (34)

where $J_{x}\left(z_{k}\right)$ penalizes the state error, $J_{\Delta u}\left(z_{k}\right)$ penalizes the change in control actions (i.e., control effort), and $J_{\varepsilon }\left(z_{k}\right)$ accounts for constraint violations. The weighting parameters were selected to emphasize position tracking over velocity regulation, aligning with the performance objectives of prosthetic wrist control. The prediction and control horizons were set to 10 and 5, respectively. The kinematic and dynamic models used in the MPC design were consistent with those presented in this study, starting from Equation 28, where $\ddot{\alpha }=y$ was considered, and the corresponding state-space representation was derived. In contrast, the SMC strategy was designed using a Piecewise Continuous Control (PCC) model, with the control input divided into two components: $u=u_{eq}+u_{sw}$ . The equivalent control $u_{eq}$ was derived using Filippov’s convexification and Utkin’s method (Utkin and Vadim, 2004) to ensure sliding motion, while the switching control $u_{sw}$ was formulated based on a Lyapunov function $V=\frac{1}{2}\sigma^{2}$ to guarantee attractivity to the sliding surface and minimize chattering. Although MPC and SMC demonstrated strong performance under nominal conditions, the impedance controller was specifically designed to handle external disturbances in a highly compliant mechanical system. Its ability to modulate interaction forces in real time makes it particularly suitable for soft prosthetic applications, where stability and adaptability under unpredictable loads are critical. Therefore, while MPC and SMC offer advantages in terms of settling time or control precision, the impedance controller provides superior robustness and trajectory recovery in dynamic environments.

While the MPC controller achieved the shortest settling time of 1.2 s , slightly outperforming the proposed impedance controller’s 3.1 s , the proposed impedance controller demonstrated superior performance in terms of accuracy and stability. Specifically, it achieved a significantly lower RMSE of $3.04\times 10^{-4}\text{ rad}$ compared to $6.0\times 10^{-3}\text{ rad}$ for SMC, $1.2\times 10^{-3}\text{ rad}$ for MRAC, and $2.1\times 10^{-3}\text{ rad}$ for MPC. Additionally, the steady-state error of the impedance controller was $1.25\times 10^{-4}\text{ rad}$ , which was notably lower than those of SMC and MRAC, and comparable to MPC. These results highlighted the effectiveness of the proposed impedance controller in delivering precise and stable control, even though its settling time was marginally longer than that of the MPC approach. While the proposed impedance controller achieved a settling time of 3.1 s under nominal conditions and up to 8 s under disturbance scenarios, the duration reflected a deliberate trade-off between response speed and compliance. In tendon-driven soft prosthetic wrists, rapid actuation can compromise system stability and user safety, particularly during interaction with unpredictable external forces. The controller was designed to prioritize smooth trajectory recovery and robust force modulation, which were essential for intuitive and safe operation in real-world prosthetic applications. Although alternative controllers such as MPC, SMC, and MRAC demonstrate shorter settling times (ranging from 1.2 to 2.8 s), they did not match the impedance controller’s performance in terms of RMSE and steady-state accuracy. These metrics were critical for ensuring precise motion tracking and minimizing long-term drift.

### 4.2 Experimental validation and conclusion

**FIGURE 13:**
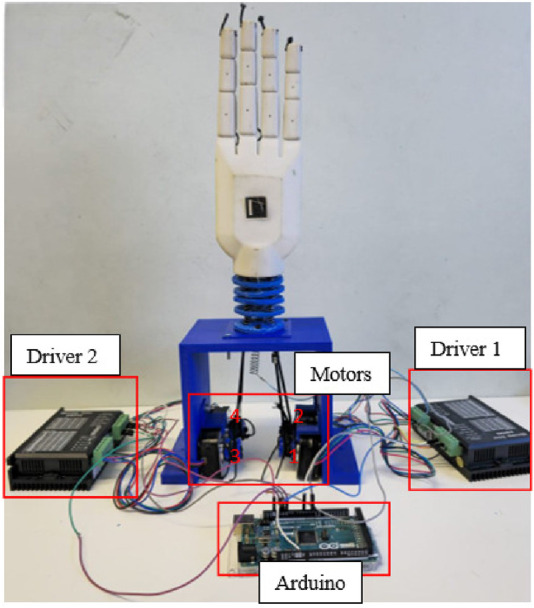
Fabricated model.

**FIGURE 14:**
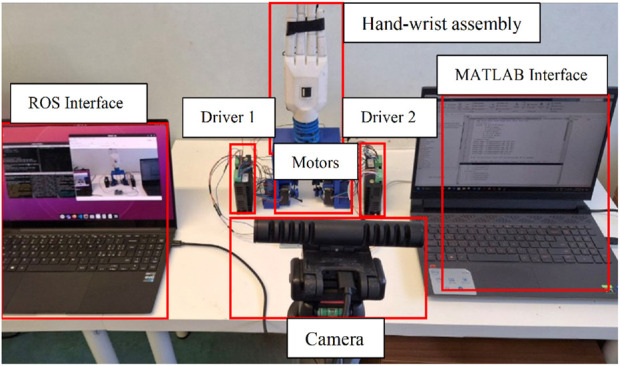
Experimentation setup.

**FIGURE 15:**
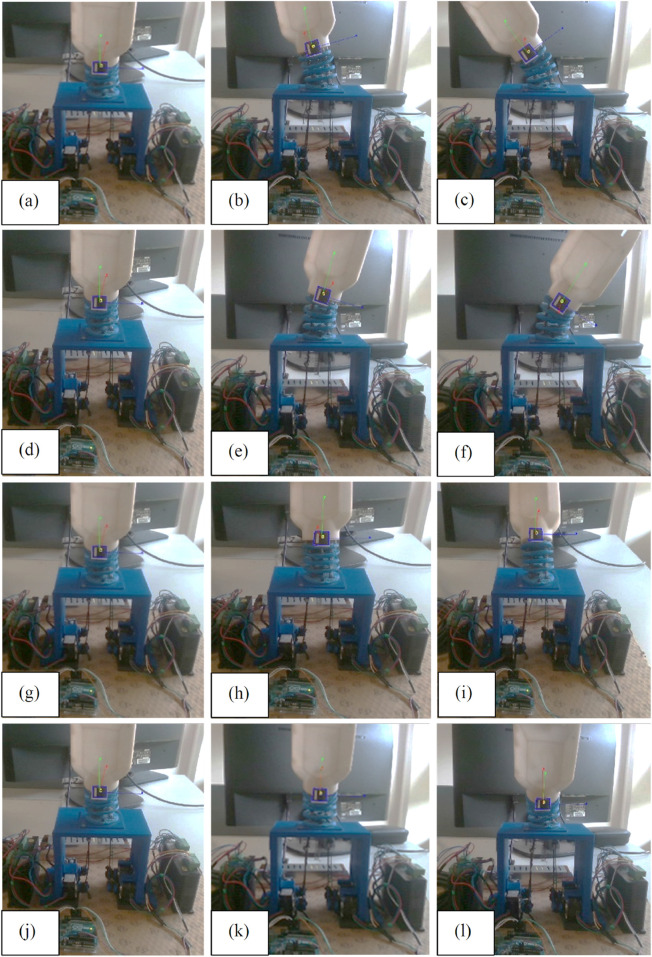
Motions of hand during experimentation **(a–c)** Ulnar **(d–f)** Radial **(g–i)** Flexion **(j–l)** Extension.

**FIGURE 16:**
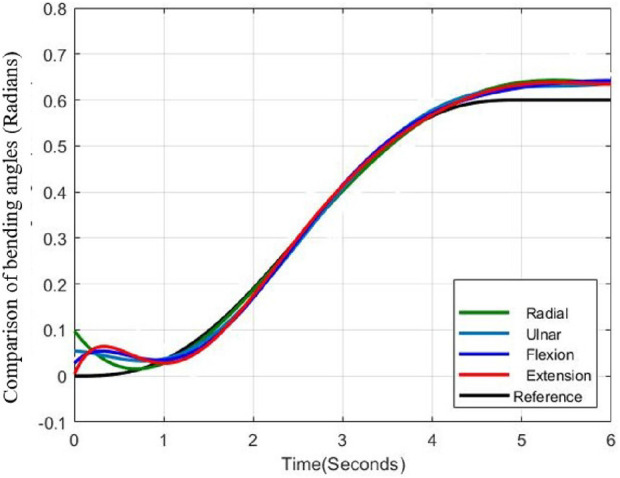
Comparison of bending angles during experimentation.

The fabricated model of the wrist section integrated with prosthetic hand is shown in Figure 13 and experimental set up is depicted in Figure 14. An ArUco marker attached to the hand enabled the tracking of its positions throughout the experiments. The setup comprised four stepper motors, two motor drivers, a 3D depth camera, and an Arduino controller for real-time functionality. Furthermore, ROS and MATLAB softwares were utilized for tracking the ArUco poses and implementing the control scheme, respectively. Tendons one and two were engaged for radial deviation of the wrist, while tendons four and five managed movements in the ulnar direction. Additionally, tendons one and four were responsible for extension motions, whereas flexion was governed by tendons two and 5. The lowest disc (disc 1) was secured to a stable platform, and the highest disc (disc 5) was connected to the hand. Motions in all directions are illustrated in Figures 15a - l, while the trajectories associated with these motions are presented in Figure 16. During the experimentation, the average RMSE values for deflection, settling time, and steady-state error across all directions were recorded as $2.7\times 10^{-2}\text{ rad}$ , 4.35 s, and $1.8\times 10^{-2}\text{ rad}$ , respectively. The findings clearly indicated that the error values observed during the experimental phase were significantly greater than those recorded in the simulation study. A primary factor contributing to the increased error margin in the experimental phase was the lower stiffness of the springs used in the wrist segment. we have also assessed the adaptability of the controller under the influence of external unknown forces.

**FIGURE 17:**
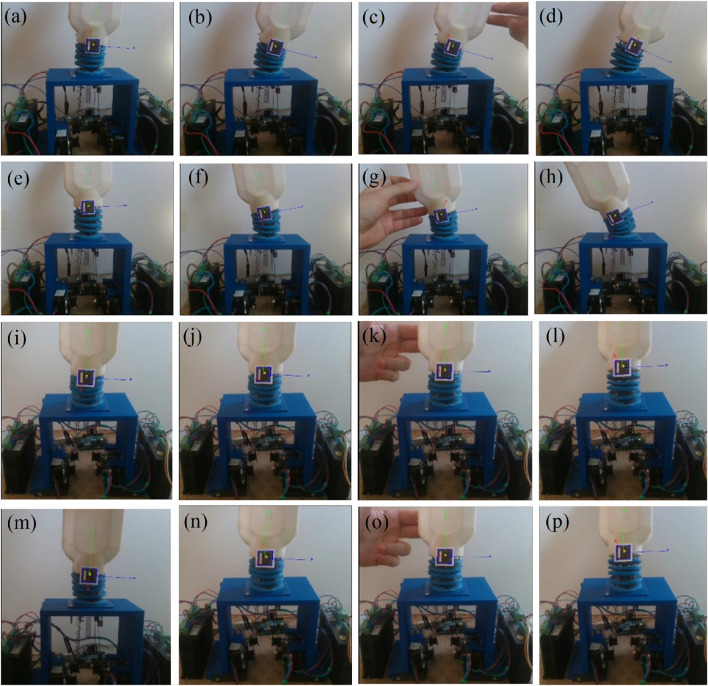
Motions of hand in presence of force during experimentation **(a–d)** Ulnar **(e–h)** Radial **(i–l)** Flexion **(m–p)** Extension.

**FIGURE 18:**
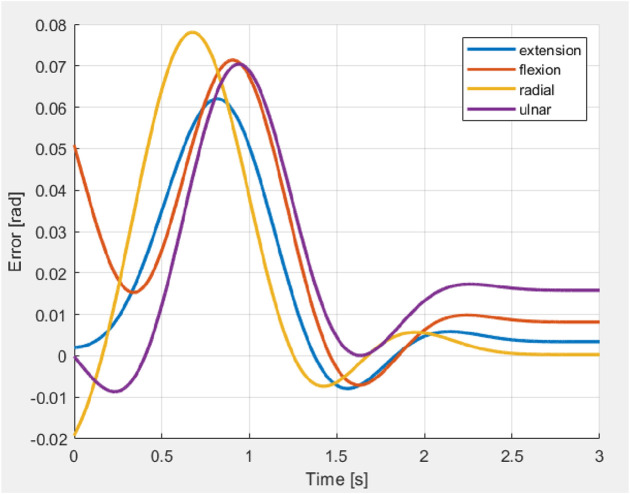
Variation of error during application of external force in four directions.

Experimental validations were carried out by applying external unknown forces by pulling the hand to the opposite directions while the hand is moving in various directions as shown in Figure 17. An external force was exerted on the hand to shift it in the opposite direction (at 0.5 s) and after the application of the external force the hand retained the trajectory and reached the desired bending angle (0.6 rad) as shown in Figure 18.

During the application of a constant external force, the system exhibited a RMSE of $1.07\times 10^{-1}\text{ rad}$ , a settling time of 2.62 s , and a steady-state error of $0.03\times 10^{-1}\text{ rad}$ . Although the RMSE, settling time, and steady-state error values observed during the application of force were slightly higher compared to the nominal case, all values remained well within acceptable tolerance limits. This indicates that the proposed impedance controller maintained robust performance even under external disturbances. During experimental validation, the controller consistently ensured convergence of the error, demonstrating its effectiveness in preserving system stability and accuracy in the presence of external forces.

Across all trials, the system’s position consistently exceeded the reference signal, a behavior attributed to the high mechanical compliance of the structure and progressive spring degradation resulting from repeated experimental cycles. The phenomenon suggested that the system occasionally failed to maintain its intended position, leading to minor bending effects. In all cases, the error peak remained within an acceptable range, reaching a maximum of 0.08 rad. Notably, the oscillatory behavior observed in simulation was absent in the experimental data, primarily due to the filtering of the control signal prior to actuator input, as previously discussed. The force was introduced during the transient phase, consistent with the conditions used in simulation. A minor undershoot observed at approximately 1.5 s indicated a rapid recovery of the system once the external force was removed.

## 5 Conclusion and scope for future work

This study presented a novel learning-based impedance control strategy for a tendon-driven soft continuum wrist integrated with the PRISMA HAND II prosthetic system. By employing an NN to estimate nonlinear impedance components, and modeling the wrist using Euler-Bernoulli beam theory and the Euler-Lagrange method, the controller effectively addressed the challenges of compliance, adaptability, and nonlinear dynamics inherent in soft prosthetic systems. Simulation studies demonstrated high accuracy with low RMSE values, minimal steady-state errors, and efficient settling times. Experimental validation confirmed the controller’s robustness in the presence of external disturbances and variations in system parameters, although performance slightly decreased due to mechanical limitations in hardware.

This research introduced a novel impedance control framework that integrates neural network-based prediction within a physics-grounded model of a soft continuum wrist for prosthetic applications. Across six simulated scenarios including nominal settings, variable spring stiffness, and external force applications the controller consistently achieved RMSE values ranging from $2.99\times 10^{-4}$ rad to $4.25\times 10^{-4}$ rad, settling times between 3.1 s and 8 s, and steady-state errors under $2.04\times 10^{-4}\;rad$ , showcasing its adaptability and precision. Experimental trials, despite increased mechanical uncertainties, demonstrated an average RMSE of $2.7\times 10^{-2}$ rad, convergence within 4.35 s, and steady-state error of $1.8\times 10^{-2}$ rad, affirming the controller’s robustness in real-world conditions. Notably, under constant external force, the system maintained stability with an RMSE of $1.07\times 10^{-1}$ rad and returned to trajectory within 2.62 s. When benchmarked against SMC, MRAC, and MPC approaches, the proposed strategy achieved the lowest RMSE and competitive steady-state accuracy, underscoring its advantage in managing soft prosthetic systems with high nonlinearity and external perturbations.These findings support the viability of integrating machine learning with physics-based modeling to develop intelligent prosthetic control systems that offer more natural and responsive movement. The proposed controller marks a significant step toward bridging the gap between rigid control mechanisms and the nuanced demands of soft prosthetics. Future work may focus on enhancing the mechanical properties of the wrist structure, expanding user adaptability through closed-loop human feedback, and implementing the controller in broader wearable and assistive robotics platforms. To address the concern regarding response speed, future work will focus on optimizing the controller’s dynamic parameters such as stiffness and damping gains, and exploring hybrid control strategies that integrate predictive or adaptive components. These enhancements aim to reduce settling time while preserving the compliance and robustness that define the impedance control framework.

## Data Availability

The original contributions presented in the study are included in the article/supplementary material, further inquiries can be directed to the corresponding author.
